# Enhancing High-Speed Penetration Resistance of Ultra-High-Performance Concrete Through Hybridization of Steel and Glass Fibers

**DOI:** 10.3390/ma18122715

**Published:** 2025-06-09

**Authors:** Mehmet Gesoglu, Guler Fakhraddin Muhyaddin, Yavuz Yardim, Marco Corradi

**Affiliations:** 1 Independent Researcher, Gaziantep 27440, Türkiye; mgesoglu27@gmail.com; 2 Construction and Materials Technology Department, Erbil Polytechnic University, Erbil 44001, Iraq; guler.muhyaddin@epu.edu.iq; 3 Department of Civil and Environmental Engineering, The University of Edinburgh, Edinburgh EH9 3FB, UK; yyardim@ed.ac.uk; 4 Department of Engineering and Technology, University of Huddersfield, Huddersfield HD1 3DH, UK

**Keywords:** ultra-high-performance concrete, steel fibers, glass fiber, penetration resistance

## Abstract

Ultra-high-performance concrete (UHPC) is a material with high mechanical properties that requires the use of fibers to overcome its brittleness, but the use of only one type of fiber may not improve UHPC performance enough. This study investigates the hybrid use of steel and glass fibers to achieve ultra-high strength along with improved ductility and impact resistance. A total of 22 concrete samples, including both plain (unreinforced) and fiber-reinforced types, were produced using micro straight-steel fibers, hooked steel fibers, and micro glass fibers, either individually or in combination. The mechanical properties, ductility, and impact behavior of the concrete samples were evaluated through experimental testing. The inclusion of microfibers had little impact on the compressive strength of concrete, which remained in the range of 130–150 MPa. However, it significantly enhanced the tensile strength, as evidenced by a flexural strength increase of up to 163% compared to the control concrete without microfibers. Numerical simulations were carried out to complement and validate the experimental investigation of projectile penetration. The depth of projectile penetration (DOP) test results were compared with existing empirical models from the literature. The incorporation of hooked steel fibers in hybrid blends significantly improved ductility and enhanced penetration resistance. In addition, previously proposed models from the literature were found to be highly conservative in predicting DOP at high projectile velocities.

## 1. Introduction

Ultra-high-performance concrete (UHPC) typically exhibits compressive strength above 150 MPa and flexural strength exceeding 15 MPa, making it significantly stronger than conventional concrete. To achieve such a high strength, a very dense matrix with minimal pore volume is required. Even though the high cementitious material content in the mixture provides ultra-high strength, the concrete becomes highly prone to cracking. To address this, fibers are incorporated into plain UHPCs to improve crack control and toughness, resulting in what is referred to as ultra-high-performance fiber-reinforced concrete (UHPFRC), a material known for both its exceptional strength and enhanced ductility [1,2,3].

Fibers improve concrete performance primarily through mechanical anchorage, which requires high stiffness, tensile strength, and strong bonding with the matrix. In addition to the aspect ratio (length/diameter), shape and surface texture are also important parameters for the bonding of fibers to the concrete matrix. Hooked steel fibers with an aspect ratio of 80 and a length of 30 mm have been shown to provide better tensile and fracture performance than micro steel fibers [3,4,5]. However, incorporating microfibers yields higher compressive strength [6,7]. A single fiber type typically enhances only a few properties, whereas combining different fibers—referred to as hybrid fibers—can improve multiple performance aspects of concrete [8,9,10]. For instance, among four fiber types—twisted steel, hooked steel, polyethylene (Spectra), and polyvinyl alcohol (PVA)— twisted fibers provided the highest flexural strength due to their superior anchorage with a concrete matrix [11]. Additionally, the hybrid use of long and medium-length straight steel fibers was found to significantly enhance the flexural performance of UHPC in terms of post-cracking strength, deflection capacity, and energy absorption, while combinations of long and short fibers were less effective [12]. Niu et al. [13] reported that a mixture of medium-length fibers and long fibers gave the highest flexural strength to UHPCs with a 2% fiber volume. Chun and You [14] investigated the dual use of both macro steel fibers and micro steel fibers. Their research showed that the hybrid use of hooked and twisted fibers resulted in improved fiber efficiency.

UHPC reinforced with fibers offers high impact resistance, making it interesting for demanding structural applications [15,16]. However, key research gaps remain, including limited understanding of the combined effects of different fiber types and dosages on impact behavior, insufficient long-term performance data, and a lack of standardized testing methods. Addressing these gaps is essential to optimize UHPC’s protective capabilities. These types of reinforced concrete have been explored in several experimental studies, particularly under projectile loading conditions [17,18]. Wu et al. [15] conducted projectile impact tests on concrete targets manufactured using medium-length steel fibers.

In their study, a UHPC with a nominal compressive strength of 90 MPa and 1.5% of the steel fiber content used was found to be among the most efficient and, at the same time, the most economical options for concrete to be used in protective structural systems. Feng et al. [16] investigated the penetration resistance of an ultra-high-performance hybrid fiber-reinforced cementitious composite with a 2% fiber content. The fibers were selected for cementitious composite strengths ranging from 70 to 115 MPa compressive strength, and when fibers were classified according to their chemical structure and size, the groups were polypropylene, polyvinyl alcohol, and micro steel fibers. The capacity developed against penetration showed a positive effect in the series formed with both steel fibers and polypropylene fibers, while it showed a negative effect in cementitious composites using steel fibers and polyvinyl alcohol fibers.

The blast and penetration resistance of UHPCs were investigated in a state-of-the-art investigation [17]. In another study, it was shown that although the increase in fiber volume fraction had a limited effect on penetration depth, it effectively reduced the crater area and volume [18]. UHPC has been reported to be an effective material for reducing the penetration depth of projectiles depending on the fiber used as well as the compressive and flexural strengths. Liu et al. [19] conducted a study on the impact responses of UHPC targets with 3% polyethylene or steel fibers when subjected to projectile strikes at velocities up to 800 m/s. Liu et al. [20] also presented a numerical study evaluating the projectile penetration into UHPC cylinders with a broad striking velocity. They recommended a modified empirical formula to predict the depth of penetration (DOP) based on experimental results. Estimating DOP using existing empirical models was reasonable for a low impact velocity and a normal concrete compressive strength. Similarly, Wu et al. [21] indicated failure of their DOP prediction model under high-speed projectile impacts. It is clear that previously proposed models become quite conservative with increasing velocity of the projectile and/or compressive strength of the concrete target. Therefore, the literature on the performance of previous models in predicting the depth of penetration indicates the need for further experimental and numerical research, particularly focused on ultra-high-performance fiber-reinforced concrete targets subjected to high-speed projectile impacts. However, the combined use of micro steel, hooked steel, and glass fibers has not yet received sufficient attention regarding the reinforcing, toughening, and impact resistance of UHPCs.

## 2. Experimental Study

### 2.1. Materials and Concrete Mixture Proportioning

**Figure 1:**
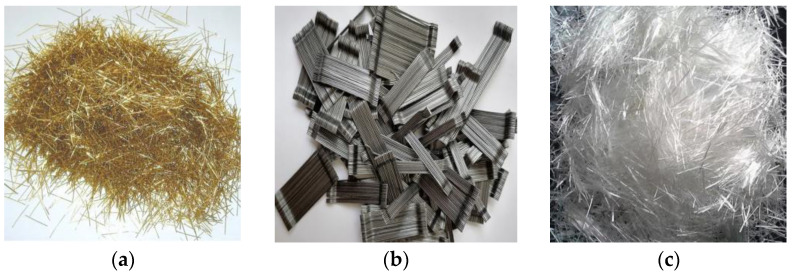
The different types of fibers employed in this study: (**a**) Brass coated micro steel fiber; (**b**) stainless steel hook-end steel fiber; (**c**) glass fiber.

Ultra-high-performance concretes were produced using an ASTM C150 [22] Type I Portland cement and silica fume as the cementitious materials. The specific gravity of the cement is 3.15, while the silica fume has a specific gravity of 2.2. Additionally, the specific surface area of the cement was 394 m^2^/kg, whereas the silica fume had a specific surface area of 21,080 kg/m^3^. The aggregate used was locally available in the Aegean region of Turkey, made of quartz, with a density of 2.65. The control mix was designed as plain concrete containing 940 kg/m^3^ cement and 240 kg/m^3^ silica fume at a water/binder ratio of 0.195. UHPFRCs were produced by adding micro steel fiber (MSF), hooked steel fiber (HSF), and glass fiber (GF) to concrete at volumetric ratios ranging from 0.25% to 2%. The selected mixing ratios were 0.25, 0.5, 0.75, 1, 1.50, and 2%. The properties of the fibers, according to the producer data sheets, are presented in Table 1, while their images are displayed in Figure 1.

Table 2 shows the concrete mixture proportioning of plain concrete as the control one and a total of 21 fiber-reinforced samples. For the workability test, varying proportions of Hyper-Plasticizer (HP), BASF, Ludwigshafen, Germany were used, taking into account the mix properties, and we aimed to achieve a flow radius of 23 ± 2 cm. The samples investigated herein were adopted from studies conducted in the same laboratory center because the same materials were previously used in concrete production [23,24]. Table 2 presents the mix details for the samples used in this study. The fibers included in the mix are named according to their type and volume content. For example, MSFHSF2.00 stands for a fiber-reinforced mix consisting of 1% micro steel fiber (MSF) and 1% hooked steel fiber (HSF).

Mixing of the UHPFRCs was conducted using a Hobart mixer, Hobart Corporation, Troy, MI, USA as described in [1,23,24]. Before preparing the mix, the aggregates were mixed as a dry mix in the mixer for 3 min; then, the aggregates were wetted using half the amount of water and re-mixed at the same speed of 100 rpm for 5 min. The superplasticizer and the remaining amount of water were then added to the pre-mixed mixture and the mixing process was repeated at 470 rpm for about 5 min. The newly formed concrete was then poured into steel molds and compacted by vibration. Finally, the specimens were placed in the molds and stored under polyethylene sheets at 22 °C for 16 h. After demolding, the specimens were immersed in water for 90 days for curing.

### 2.2. Testing Methods

The following sections briefly outline the testing methods used for the three types of experiments conducted: compression, bending, and projectile penetration tests performed on the samples.

#### 2.2.1. Compressive Testing

Compressive strength was evaluated on 100 mm cubes at 90 days after casting. The tests were conducted using a testing machine under load control at a rate of 200–300 kN/min. A 2000 kN load cell concrete press was used in the laboratory, with the cubic samples placed directly between the press’s steel plates. Testing was halted when a 20% drop in load capacity was detected by the machine. Compressive strength was calculated by dividing the maximum applied load by the loaded area of the specimens (100 × 100 mm).

#### 2.2.2. Bending Testing

**Figure 2:**
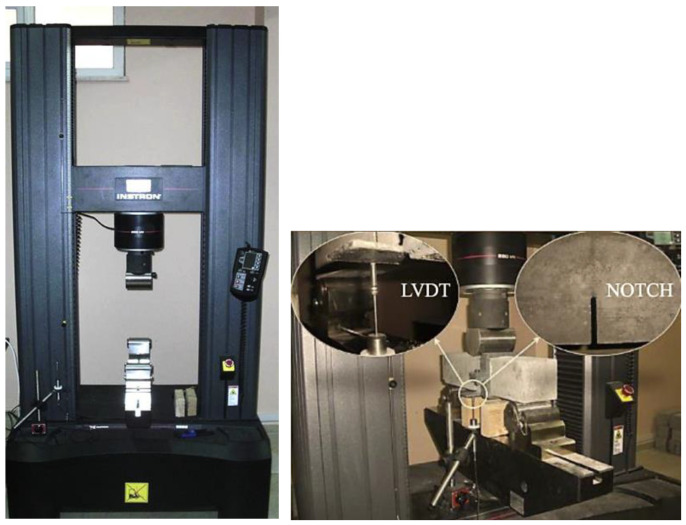
Three-point bending testing machine and test specimen [25] used for calculation of modulus of rupture.

Bending tests were carried out using a three-point bending configuration on notched beams measuring 100 × 100 × 500 mm. The tests were conducted with a closed-loop testing machine under displacement control at a rate of 0.02 mm/min, as illustrated in Figure 2. Testing was conducted in accordance with guidelines of the Technical Committee of RILEM 50-FMC/198 [25]. For this, two electronic dial gauges were fixed on the sides of the beam. The specimens to be tested were first notched to a depth of 40 mm using a saw. Two 90-day old beams from each mix were tested to determine their fracture energy (*G_F_*) and modulus of rupture (*f_flex_*):

(1) $$G_{F}=\frac{W_{o}+mg\delta_{s}\frac{S}{U}}{B\left(W-a\right)}$$

(2) $$f_{flex}=\frac{3P_{max}S}{2B{\left(W-a\right)}^{2}}$$

where *W_o_* is the area underneath the load–deflection curve; *m* is mass of the concrete beam; *g* is the gravitational acceleration; *δ_s_* is target displacement; *S* is the span; *U*, *B*, and *W* are the length, width, and depth of the concrete beam; *a* is the notch depth; and *P_max_* is the peak load.

#### 2.2.3. Penetration Testing

**Figure 3:**
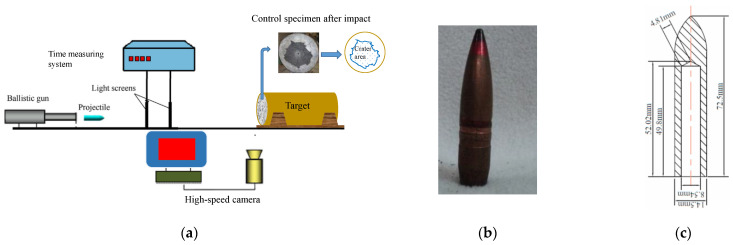
(**a**) Test setup for projectile impact and surface damage of plain control concrete; (**b**) photo of the projectile; (**c**) projectile dimensions.

The penetration test setup is shown in Figure 3. The projectiles used were made of common steel with a nominal yield strength of 800 MPa. Ogival nose-type projectiles with a 14.5 mm diameter, a 3.0-caliber radius head, and a 54.2 g weight were launched by a ballistic gun with adjusted propellant powder to provide a striking velocity of approximately 1050 m/s. It was measured by means of a velocity measuring device instrumented with two light curtains 2 m apart and an electronic time recorder. The average velocity of the projectile passing through the light curtains was assumed to be the approximate impact velocity. Furthermore, the utilization of a high-speed camera allowed for the capture of the impact position. For each concrete mixture in Table 2, three concrete cylinders with a 400 mm diameter and a 400 mm height were cast into 3 mm thick steel culvert molds by following the mixing procedure above. Because the shank diameter of the projectile was almost 27 times smaller than the target diameter, the lateral confinement effect of the steel culvert was negligible, as recommended by Feng et al. [16]. Similarly, Wu et al. [15] selected a target diameter almost 29 times the projectile diameter so that the circumferential boundary effects could be negligible. The DOP was measured, and the area of the crater on the damaged surface was computed after the target was shot. The inset of Figure 3 illustrates the damaged surface accompanied by a schematic of the crater area of the plain control concrete.

#### 2.2.4. Numerical Modeling

**Figure 4:**
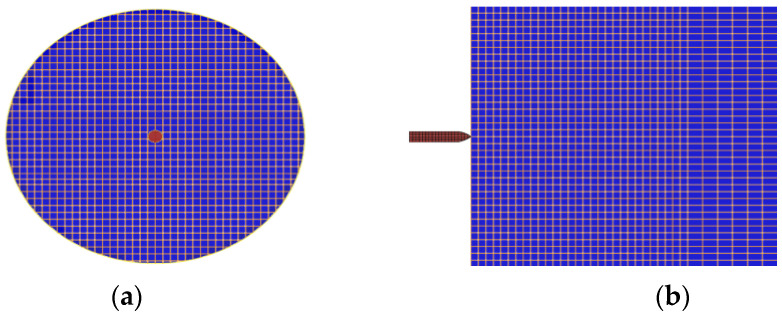
Penetration modeling and the mesh; (**a**) front view (**b**) side view.

Penetration of the projectile into the UHPFRC was simulated via the LS-DYNA explicit finite element program (Version 971 release 5266), in which the concrete target and the steel projectile were modeled as separate parts. Figure 4 shows the grid of the interaction area between the missile body and the target cementitious material. Although LS-DYNA (offers several concrete material models, the Concrete Damage Rel3 model (MAT_72 REL3) was selected to represent the target concrete, in combination with MAT_ADD_EROSION and the EOS_TABULATED_COMPACTION equation of state. Although the numerical modeling procedure is relatively simple, the models used in this simulation—validated in previous studies—have at times demonstrated reliable predictions of the structural response of reinforced concrete with microfibers. This is primarily because many of the material properties and parameters—such as compressive strength, weight density, and Poisson’s ratio—used in the equation of state (EOS Tabulated Compaction) model allow for automatic calculations [26,27,28]. The relevant input parameters for MAT_72 REL3 were modified using the concrete density values in Table 2, the compressive strength and modulus of rupture in Table 3, a Poisson’s ratio of 0.19, a characteristic element size (h) of 2.8 mm, a localization width (*LocWidth*) of 1.9 mm, the parameters related to a porosity compaction curve (b_1_ and b_2_) of 0.82, and a b_2_ value between 0.45 and 1.42. b_1_ and b_2_, respectively, govern the compressive and tensile softening damage evolution, as the stress point moves from the maximum-strength surface to the residual-strength surface in the stress-and-strain curve. The shear dilatancy factor, Omega, which governs volumetric expansion, was set to 0.75, following the recommendation of Wu et al. [29]. An automatically generated equation of state, EOS_TABULATED_ COMPACTION, with a calibrated bulking unloading modulus was applied to reflect the volumetric stress and strain with respect to the impact response caused by high-velocity projectile penetration. Finally, an erosion algorithm, MAT_ADD_EROSION, was also employed in the material model to avoid element distortion during the simulation. When the defined criterion reached the critical value, the failed element was discarded from the calculations. Therefore, a maximum principal strain of 0.16 was chosen as the erosion criterion in the numerical simulation for the conservative calculation, as proposed in the literature [30,31].

Furthermore, in concrete modeling, a numerical erosion algorithm, known as Mat Add Erosion, was used to prevent element distortion during the simulation. For this purpose, the fracture and failure of the material and stresses under compression and shear were defined as the two main exceedance criteria. In fact, when the critical threshold between the defined criteria was reached, the fractured element was immediately removed from the numerical analysis. In this study, a conservative estimate for the values of both compressive and shear stresses was made and set at a normalized value of 0.2.

**Figure 5:**
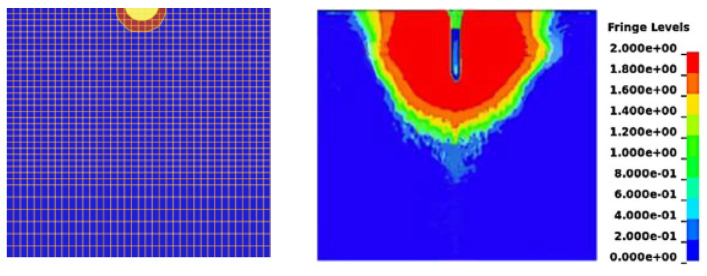
Details of the mesh used in the numerical model and an illustration of plastic strain development [Von Mises stress contour (unit: 10^11^ Pa)].

The material model of Mat Johnson Cook together with EOS Gruneisen (equation of state) [32] was employed to model the casing and backfill of the steel projectile. It has a steel casing with a nominal weight density of 7850 kg/m^3^. The Contact_Eroding_Surface_To_Surface definition was used for the interaction between the projectile and the UHPC target. The modeling results show that the boundary conditions have a negligible influence on concrete targets because the projectile diameter is much smaller than the target [19]. Figure 5 illustrates the mesh used in the numerical model for the penetration testing, and the strain development in the concrete target.

## 3. Result and Discussion

### 3.1. Compressive Strength

**Figure 6:**
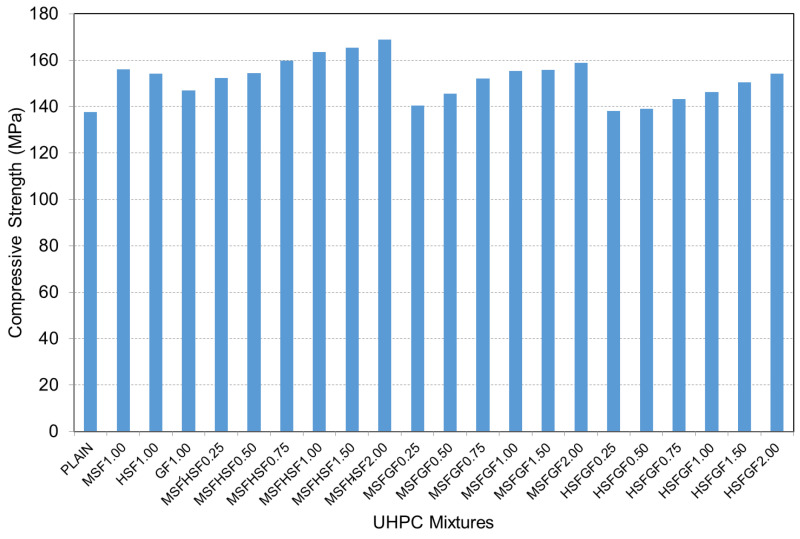
Compressive strength of concrete mixtures with hybrid fibers.

**Figure 7:**
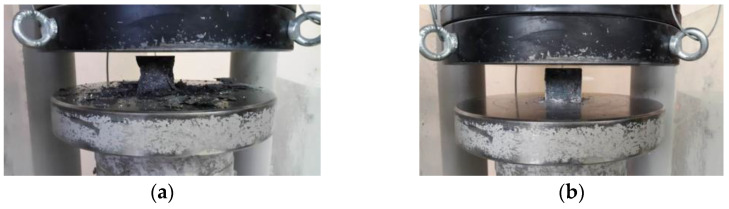
Compressive testing: (**a**) plain specimen; (**b**) fiber-reinforced specimen.

The variation in compressive strength between plain and fiber-reinforced UHPCs is displayed in Figure 6. The addition of hybrid fibers to the mixtures led to a significant enhancement in compressive strength. The plain concrete had a compressive strength of about 137 MPa, increasing to almost 169 MPa in the case of MSFHSF concrete. Although the strength increases were smaller than those observed in the MSFHSF mix, the compressive strength of MSFGF was approximately 159 MPa, while for HSFGF mixtures at a 2% fiber volume, this value was 154 MPa. When a single type of fiber was included in the concrete mixtures at 1% by volume, the compressive strength results for concrete mixtures containing MSF and HSF were 156 MPa and 154 MPa, respectively. It can be observed from Figure 6 that the addition of fiber reinforcement did not significantly improve the compressive strength of the concrete samples. This outcome is expected, as concrete inherently exhibits high compressive strength but relatively low tensile resistance. This characteristic is clearly reflected in the results of our experimental investigation. Figure 7 shows the failure modes of both the plain and fiber-reinforced specimens.

However, concrete mixtures containing 1% MSFHSF hybrid fiber resulted in a compressive strength of approximately 164 MPa with an increase in strength of 6%. Considering the effect of fibers on compressive strength, the fibers were ranked as follows: MSF had the most positive effect, followed by GF, while HSF had the least. The lower compressive strength of the HSF-incorporated concretes was attributed to the large quantity of hyper plasticizer (HP) used to achieve the target slump flow, which caused higher entrapped air content, which thus reduced the strength. Moreover, Gesoglu et al. [1] and Muhyaddin [22] attribute this behavior to the agglomeration of long fibers, which can be resolved by using a higher amount of HP.

### 3.2. Modulus of Rupture

**Figure 8:**
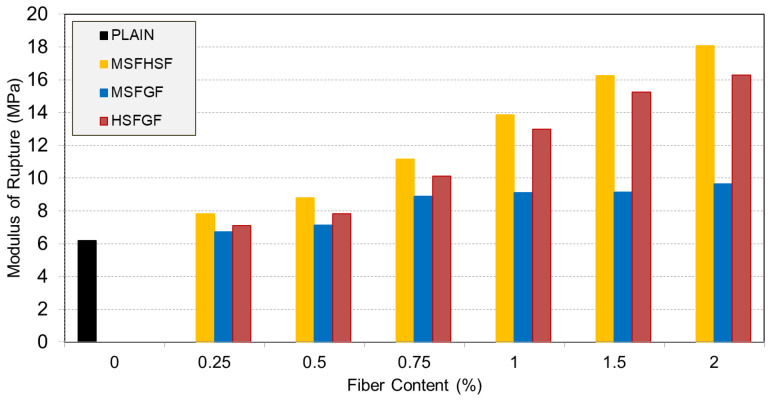
Impact of hybrid fiber content on modulus of rupture.

**Figure 9:**
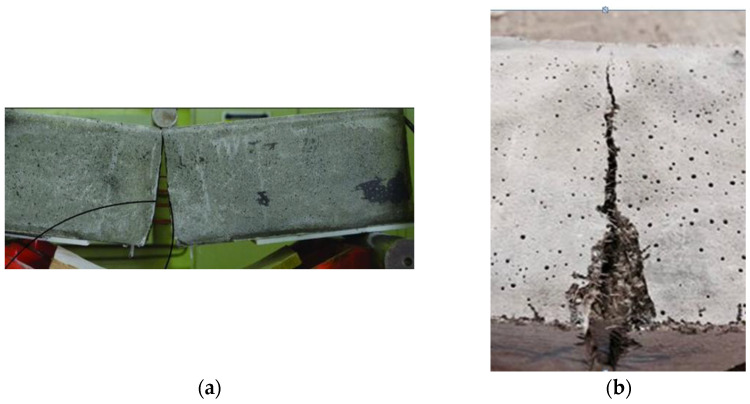
Failure modes of bending tests: (**a**) plain; (**b**) fiber-reinforced.

The modulus of rupture values, resulting from bending tests, are listed in Table 3 and graphically displayed in Figure 8. The plain concrete had a modulus of rupture of 6.18 MPa, which increased to almost 7 MPa, 12 MPa, and 8 MPa when MSF, HSF, and GF were added in the mixture at a 1% fiber volume. Regarding the mixtures of the hybrid fibers, the concretes with both types of steel fibers (MSFHSF), micro straight-steel and glass fibers (MSFGF), and hooked steel and glass fibers (HSFGF) had moduli of rupture of 13.86 MPa, 9.12 MPa, and 12.9 MPa, respectively. At a fiber volume of 1%, the use of mono-HSF improved the flexural strength by 94%. The flexural strengths experienced enhancements of 124% and 108%, respectively, when HSF was combined with MSF or GF. Considering the effect of HSF, it showed improved performance in terms of flexural strength. This improved performance was superior in terms of HSF’s ability to limit crack formation, i.e., bridging and delaying crack propagation, as well as its increased capacity to transform the fracture behavior of concrete from brittle to ductile. Furthermore, the capacity of HSF to form a good bond between the fiber and the concrete matrix, as a result of its long and hooked ends, helped to achieve the best possible performance target for flexure strength [33,34,35], as shown in Figure 9.

### 3.3. Fracture Toughness

**Figure 10:**
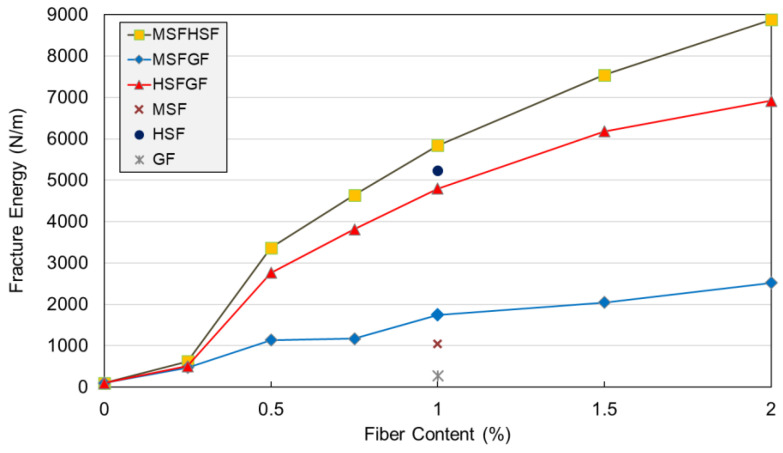
Effect of combined use of fibers on fracture energy of UHPCs.

The ductility of concrete is measured in terms of fracture energy as the area underneath the load–deflection curve. Previous studies have revealed that higher fracture toughness makes concrete more ductile. The MSFHSF mixture demonstrated the highest fracture energy, as illustrated in Figure 10. The reason for this mixture having the highest energy value is that the characteristic behavior in the load–deflection curve of such concretes leads to greater energy absorption. Indeed, the concretes with HSF had longer descending tails that led to longer softening parts and, in turn, longer tails that led to greater fracture energy. Regardless of the fiber content, HSF-reinforced concretes maintained this positive feature in all mixtures. The effect of fibers on the fracture toughness was significant. As shown in Table 3, plain concrete had a fracture toughness of 103 N/m, which increased to 1046 N/m, 5239 N/m, and 276 N/m when the MSF, HSF, and GF were used in concrete at a 1% volume. Moreover, the combined use of MSFHSF, HSFGF, and MSFGF at a 1% volume further increased the fracture toughness to 5841 N/m, 4797 N/m, and 1739 N/m, respectively. Even at a low content, using MSF or GF alongside HSF resulted in remarkable ductility. As a result, concrete matrices reinforced with HSF showed increased ductility in terms of fracture energy and flexural strength. In addition, the effect of fiber content was more significant in HSF-reinforced concretes.

### 3.4. Depth of Penetration (DOP)

**Figure 11:**
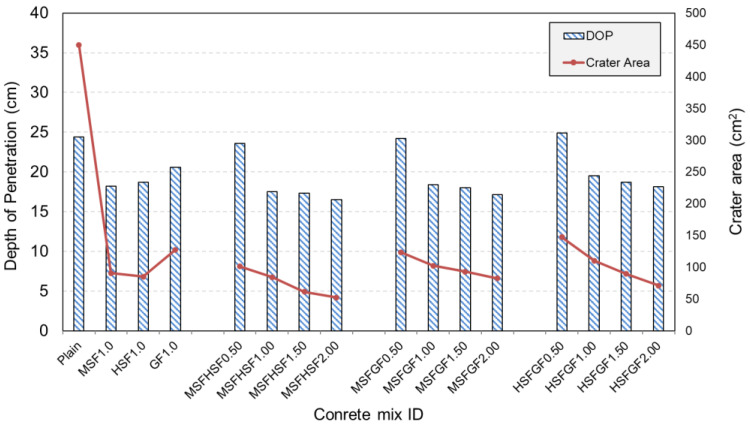
DOP and crater area of UHPFRCs.

The DOP value accounts for the actual penetration depth of a projectile, including the ballistic tunnel, as well as its penetrated length within the target. Figure 11 shows the variation in DOP values of the plain and fiber-reinforced UHPCs. An observation was made that UHPC without fiber had a DOP value of approximately 24 cm, which decreased to 18 cm and 21 cm with the use of 1% mono-steel and -glass fibers, respectively. It was evident that the steel fibers were more effective than the glass fibers in mitigating the damage induced by the projectile impact. This may be attributed to the higher compressive and flexural strengths of steel fibers and the lower pull-out resistance of glass fibers. As suggested by Wu et al. [15], the enhanced material strength due to the fiber reinforcement generated more resistance to projectile penetration. Hybrid use of the fibers, especially in the form of MSFHSF, resulted in lower DOP values for the concrete. The combined use of micro and hooked steel fibers was the most effective method; the DOP values, which were 18.2 cm and 18.7 cm with 1% MSF and HSF alone, dropped to around 16.5 cm when micro and hooked steel fibers were used together. Moreover, the 20.6 cm DOP value of 1% MGF concrete was reduced to 18 cm and 19 cm when the glass fibers were used in the hybrid system with MSF and HSF, respectively. The beneficial effect of fibers, especially in the hybrid form, was more favorable at higher fiber fractions.

This research demonstrates that adding microfibers to UHPC does not significantly increase its compressive strength, while its flexural strength improves substantially—up to 160%—with steel fibers showing a greater effect than glass fibers. Additionally, we observed up to a 32% reduction in DOP from projectile impact. Compared to previous studies on hybrid fiber-reinforced UHPC, such as those combining micro steel fibers with high-strength steel fibers, our DOP reduction aligns well with reported values, which typically range between 10 and 20% [36,37]. This suggests that the synergy between different fiber types in hybrid systems contributes effectively to impact resistance, consistent with prior literature emphasizing hybridization benefits. Our findings support earlier conclusions that hybrid fiber reinforcement enhances toughness and energy absorption more than single-fiber systems. However, the modest DOP reduction also indicates that while hybridization improves impact resistance, the effect may be limited by fiber dispersion and matrix–fiber bonding efficiency. Thus, our results confirm the positive synergy of fiber hybridization but highlight the need for further optimization to maximize protective performance under projectile impact.

### 3.5. Crater Area

One of the best measures to quantitatively evaluate the level of damage induced by projectile impact is the crater area [15,16,18]. In this study, the contour lines of both the target and the damaged zone were imported into AutoCAD (Version 2015, Computer software, Autodesk Inc.) to calculate the crater area. Figure 3 presents an example of the determination of the crater area for the reference concrete.

**Figure 12:**
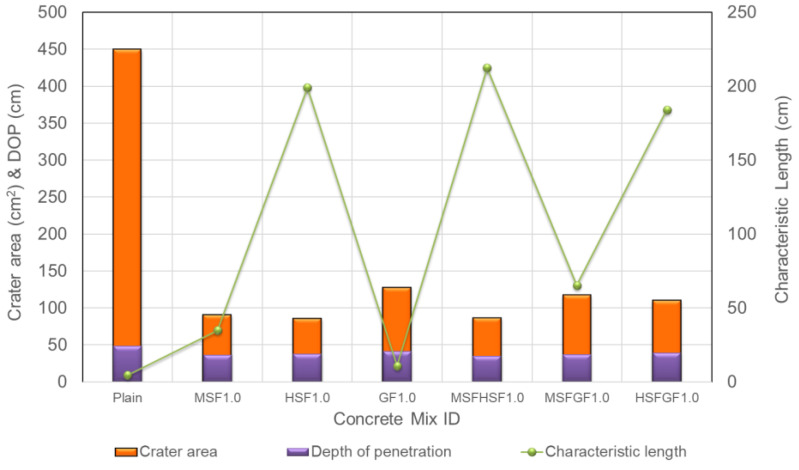
Relationship between DOP and crater area of UHPFRCs.

**Figure 13:**
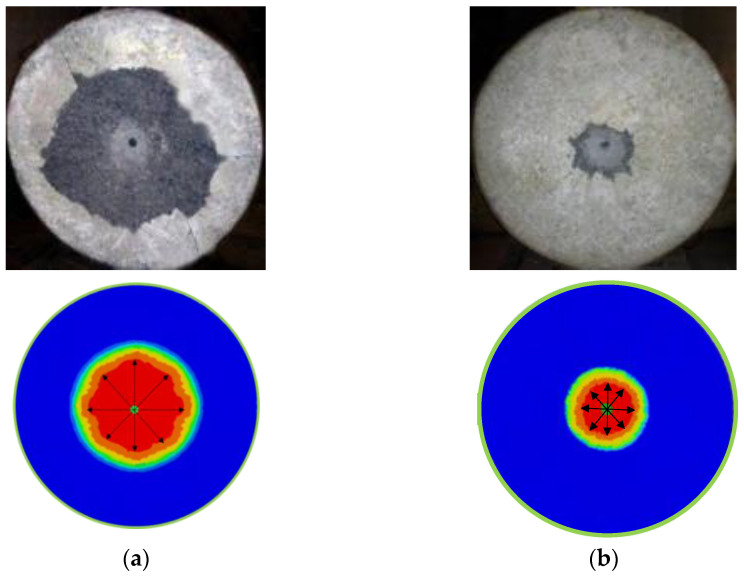
Damage levels: (**a**) plain (control); (**b**) MSFHSF2.0.

Figure 12 shows the estimated crater areas of all concrete samples. Moreover, the effect of fiber type and combination on the crater area of plain and UHPFRC targets with a 2% fiber volume is visually presented in Figure 13. The effect of fiber use in different fractions, as well as with size and type, was more favorable on the crater area than the depth of penetration. It was evident that the largest crater area of 449 cm^2^ belonged to the plain concrete, while fiber incorporation substantially reduced the crater area. The concretes with MSFHSF2.0, MSFGF2.0, and HSFGF2.0 had crater areas of approximately 52 cm^2^, 82 cm^2^, and 71 cm^2^; thus, such values indicated 88, 81, and 84% smaller crater areas, respectively, than the plain concrete. Long fibers with hooked ends, i.e., HSF, generated considerably higher bond strength between the fiber and the surrounding matrix; thus, fiber pullout without breakage could not occur. Irrespective of the fiber content, concrete with HSF remained almost intact after projectile impact, owing to good bonding generated between the long steel fibers and the concrete. Figure 12 also illustrates the relationship between the crater area, DOP, and fracture toughness. It was seen that the effect of fiber use, especially in the dual system, was more pronounced on the crater area than on the depth of penetration. Moreover, the fracture toughness of the concretes remarkably influenced the crater area, while it was less effective on the DOP values. Therefore, this suggests that the crater area is strongly correlated with the characteristic length and, in turn, fracture toughness as a measure of ductility. However, the DOP was found to be more dependent on the resulting mechanical properties, particularly the compressive strength. Among the concrete targets tested, the targets using a hybrid of MSF and HSF in their mix emerged as the best combination in the study, with a much smaller crater area and a lower calculated DOP value. This is likely due to the fact that steel fiber reinforcement increases the material’s ductility, which is the main rationale behind the experimental results of the study.

### 3.6. Analysis of Numerical Modeling and DOP Prediction by Empirical Models

**Figure 14:**
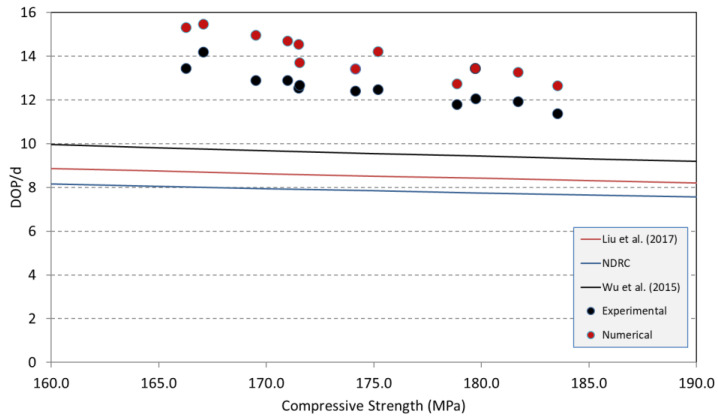
Test and simulation data compared with prediction of empirical models (d is the bullet diameter) [15,20].

Figure 14 shows a comparison between the experimental and numerical results of localized crater damage on plain and fiber-reinforced samples. Interestingly, the numerical simulation aligned well with the experimental results, as the crater area in plain concrete was much larger, and both the crater area and diameter decreased with the incorporation of fibers. Under the same striking velocity, the damage region in steel fiber-reinforced concrete targets was effectively restrained in comparison with the concrete target reinforced with glass fiber. In particular, hooked steel fibers seem to significantly reduce the crater area. This improvement can be attributed to the higher fracture toughness, which corresponds to a greater characteristic length, demonstrating that concrete reinforced with hooked steel fibers exhibits better impact resistance.

The numerical DOP values appeared to be more conservative compared to the experimental values. Indeed, the relative error in simulating the plain concrete target was only 3%, increasing to 13% for the fiber-reinforced concrete targets. These small error values demonstrate the good effectiveness of the numerical model in capturing the structural behavior of fiber-reinforced materials. However, several limitations should be considered, and the results should be interpreted with caution. Therefore, the numerical modeling shows fairly good agreement with the experimental results. The experimental (*E*) and numerical (*N*) values have a correlation, as shown in Equation (3), with a correlation coefficient (R^2^) of 0.90. Both *E* and *N* are in cm.

(3) $$E=4.53e^{0.073N}$$

There are various empirical models to predict DOP values in the literature [15,20,21,38,39,40]. The data obtained in the experimental and numerical parts of this study were compared with the estimated values of the models proposed by the US National Defence Research Committee (namely, modified NDRC) [32], Liu et al. [20], and Wu et al. [21], shown in Figure 14, for a given projectile speed of 1050 m/s and concrete compressive strength ranging from 140 MPa to 180 MPa. Among all these models used, it is clearly observed that there is an increase in the CRH ratio of the DOP, and the impact velocity of the projectile increases exponentially, but the increase in the compressive strength of the UHPC target decreases exponentially. Upon increasing the compressive strength from 140 MPa to 180 MPa (Figure 14), DOP values normalized with the projectile diameter decreased gradually in line with the literature. Both the experimental and numerical values are greater than those obtained using the recommended models, which seem to be quite conservative, in descending order of modified NDRC [32], Liu et al. [20], and Wu et al. [21]. This indicates that more accurate prediction models are necessary for ultra-high-strength concretes with varying fiber contents. Although in the study of Liu et al. [20] and Wu et al. [21], their models were quite efficient in predicting the DOP values for a striking velocity up to 900 m/s, these models highly underestimated the experimental values for higher projectile speeds in this study. These findings highlight the need to revise existing models to account for higher projectile velocities (above 900 m/s) and concrete targets made with UHPFRC.4.

## 4. Conclusions

High-speed projectile penetration tests were carried out on UHPFRC targets incorporating various fiber types and hybrid combinations to assess their performance under impact velocities exceeding 1000 m/s. The experiments confirmed the effectiveness of UHPFRC in reducing both DOP and crater damage, as well as in improving overall energy absorption through enhanced ductility and fracture resistance. The main conclusions drawn from this study are as follows:

- The mechanical properties of UHPFRCs, particularly compressive strength, had a significant effect on DOP. Thus, using a combination of fibers improved the strength values, which subsequently lowered the DOP values.
- The use of both long and short steel fibers together demonstrated the most effective performance in reducing the damage caused by projectile impacts. The crater area observed in UHPFRC, particularly with steel fibers, was significantly smaller than that of plain concrete. The MSFHSF combination reduced the crater area by up to 88% and proved to be the most effective in minimizing overall damage.
- The ductility of the concretes significantly influenced the crater area, while it had a smaller effect on the DOP values. Concrete targets with higher fracture toughness exhibited lower damage. Visual inspection of the concrete targets after projectile impact showed that using hooked steel fibers substantially reduced the crater area.
- The numerical simulation of the impact process satisfactorily matched the experimental results, with an error range of 2–14%. However, earlier models found in the literature tended to be overly conservative in their predictions of DOP values. Therefore, it is essential to revise these models for high-speed projectile impacts exceeding 1000 m/s, particularly for targets composed of UHPFRCs.
- Fracture toughness can serve as a reliable indicator of ductility in UHPFRC. Using a hybrid combination of fibers significantly increased fracture toughness, leading to enhanced ductility, especially when MSF or glass fibers (GF) were used in conjunction with HSF.

This study exhibits several limitations, notably the limited sample size, the exclusive use of a single concrete strength grade, and the evaluation of only three types of microfibers, despite the broader spectrum available commercially. Nonetheless, the findings provide valuable empirical data to the sparsely investigated domain of projectile impact resistance, contributing to the advancement of knowledge in this area of materials engineering.

## Figures and Tables

**Table 1:** Physical and geometrical properties of fibers, according to producer data sheets (Beksa Steel Cord Manufacturing Inc., Istanbul, Turkey).

Types of Fiber	Diameter (D) (mm)	Length (L) (mm)	Aspect Ratio (L/D)	Tensile Strength (MPa)	Density (g/cm^3^)
Steel	0.16	6	37.5	2250	7.17
Hooked steel	0.55	30	55	1345	7.85
Glass	0.018	13	722	2000	2.6

**Table 2:** UHPC mixture proportioning [micro straight-steel fibers (MSF), hooked steel fibers (HSF), micro glass fibers (GF), silica fume (SF), superplasticizer (SP)]. In parentheses, percentages of fiber content are given.

Mixture ID	Cement (kg/m^3^)	SF (kg/m^3^)	Water (kg/m^3^)	SP (kg/m^3^)	MSF (kg/m^3^) (%)	HSF (kg/m^3^) (%)	GF (kg/m^3^) (%)	Aggregates (kg/m^3^)
Plain					0 (0)	0 (0)	0 (0)	793.7
MSF1.0	960	240	234	45	71.70 (1)	0 (0)	0 (0)	767.2
HSF1.0					0 (0)	78.5 (1)	0 (0)	764.7
GF1.0					0 (0)	0 (0)	26.0 (1)	798.8
MSFHSF0.25					8.96 (0.125)	9.81 (0.125)		757.6
MSFHSF0.50					17.93 (0.25)	19.63 (0.25)		780.5
MSFHSF0.75	960	240	234	51	26.89 (0.375)	29.44 (0.375)	0 (0)	773.8
MSFHSF1.00					35.85 (0.5)	39.25 (0.5)		767.2
MSFHSF1.50					53.78 (0.75)	58.88 (0.75)		754.0
MSFHSF2.00					71.70 (1)	78.50 (1)		726,0
MSFGF0.25					8.96 (0.125)		3.25 (0.125)	809,2
MSFGF0.50					17.93 (0.25)		6.50 (0.25)	802.5
MSFGF0.75	960	240	234	60	26.89 (0.375)	0 (0)	9.75 (0.375)	795.9
MSFGF1.00					35.85 (0.5)		13.00 (0.5)	789.3
MSFGF1.50					53.78 (0.75)		19.50 (0.75)	717.2
MSFGF2.00					71.70 (1)		26.00 (1)	696.5
HSFGF0.25						9.81 (0.125)	3.25 (0.125)	794.4
HSFGF0.50						19.63 (0.25)	6.50 (0.25)	787.8
HSFGF0.75	960	240	234	51	0	29.44 (0.375)	9.75 (0.375)	773.8
HSFGF1.00						39.25 (0.5)	13.00 (0.5)	767.2
HSFGF1.50						58.88 (0.75)	19.50 (0.75)	739.2
HSFGF2.00						78.50 (1)	26.00 (1)	711.3

When two fiber acronyms (MSF, GF, and HSF) appear in the same mixture ID, this indicates that the sample contains a combination of both fiber types.

**Table 3:** Mechanical and fracture properties of UHPFRCs.

Mixture ID	Compressive Strength (MPa)	Modulus of Rupture (MPa)	Fracture Energy (N/mm)
Plain	137.6	6.18	103.7
MSF1.00	156.1	7.01	1046.9
HSF1.00	154.2	12.19	5239.7
GF1.00	147.0	8.27	276.0
MSFHSF0.25	152.3	7.81	621.8
MSFHSF0.50	154.5	8.81	3370.6
MSFHSF0.75	159.9	11.15	4642.0
MSFHSF1.00	163.5	13.86	5841.6
MSFHSF1.50	165.3	16.25	7540.4
MSFHSF2.00	168.8	18.08	8879.9
MSFGF0.25	140.4	6.74	471.5
MSFGF0.50	145.5	7.14	1136.6
MSFGF0.75	152.0	8.90	1172.6
MSFGF1.00	155.3	9.12	1739.4
MSFGF1.50	155.9	9.15	2044.8
MSFGF2.00	158.8	9.64	2520.8
HSFGF0.25	138.1	7.10	506.7
HSFGF0.50	139.1	7.84	2765.4
HSFGF0.75	143.2	10.12	3813.7
HSFGF1.00	146.3	12.98	4797.0
HSFGF1.50	150.5	15.24	6183.3
HSFGF2.00	154.2	16.27	6917.0

## Data Availability

The original contributions presented in this study are included in the article. Further inquiries can be directed to the corresponding author.
